# Signatures of divergent anti-malarial treatment responses in peripheral blood from adults and young children in Malawi

**DOI:** 10.1186/s12936-019-2842-7

**Published:** 2019-06-24

**Authors:** Paul L. Maurizio, Hubaida Fuseini, Gerald Tegha, Mina Hosseinipour, Kristina De Paris

**Affiliations:** 1 Present Address: Department of Medicine, Section of Genetic Medicine, The University of Chicago, Chicago, IL 60637 USA; 2 Department of Genetics, University of North Carolina-Chapel Hill, Chapel Hill, NC 27599 USA; 3 Curriculum in Bioinformatics and Computational Biology, University of North Carolina-Chapel Hill, Chapel Hill, NC 27599 USA; 4 Department of Pathology, Microbiology & Immunology, Vanderbilt University, Nashville, TN USA; 5 Division of Infectious Diseases, Department of Medicine, University of North Carolina, 130 Mason Farm Rd, Bioinformatics Bldg, Chapel Hill, NC 27599 USA; 6 University of North Carolina Project-Malawi, Lilongwe, Malawi; 7 Department of Microbiology and Immunology, University of North Carolina, Chapel Hill, NC 27599 USA

**Keywords:** *Plasmodium falciparum*, Uncomplicated malaria, Heterogeneity, Cytokines, Paediatric

## Abstract

**Background:**

Heterogeneity in the immune response to parasite infection is mediated in part by differences in host genetics, gender, and age group. In infants and young children, ongoing immunological maturation often results in increased susceptibility to infection and variable responses to drug treatment, increasing the risk of complications. Even though significant age-associated effects on host cytokine responses to *Plasmodium falciparum* infection have been identified, age-associated effects on uncomplicated malaria infection and anti-malarial treatment remain poorly understood.

**Methods:**

In samples of whole blood from a cohort of naturally infected malaria-positive individuals with non-severe falciparum malaria in Malawi (n = 63 total; 34 infants and young children < 2 years old, 29 adults > 18 years old), blood cytokine levels and monocyte and dendritic cell frequencies were assessed at two timepoints: acute infection, and 4 weeks post anti-malarial treatment. The effects of age group, gender, and timepoint were modeled, and the role of these factors on infection and treatment outcomes was evaluated.

**Results:**

Regardless of treatment timepoint, in this population age was significantly associated with overall blood haemoglobin, which was higher in adults, and plasma nitric oxide metabolites, IL-10, and TNF levels, which were higher in young children. There was a significant effect of age on the haemoglobin treatment response, whereby after treatment, levels increased in young children and decreased in adults. Furthermore, there were significant age-associated effects on treatment response for overall parasite load, IFN-*γ*, and IL-12(p40), and these effects were gender-dependent. Significant age effects on the overall levels and treatment response of myeloid dendritic cell frequencies were observed. In addition, within each age group, results showed continuous age effects on gametocyte levels (*Pfs16*), TNF, and nitric oxide metabolites.

**Conclusions:**

In a clinical study of young children and adults experiencing natural falciparum malaria infection and receiving anti-malarial treatment, age-associated signatures of infection and treatment responses in peripheral blood were identified. This study describes host markers that may indicate, and potentially contribute to, differential post-treatment outcomes for malaria in young children versus adults.

**Electronic supplementary material:**

The online version of this article (10.1186/s12936-019-2842-7) contains supplementary material, which is available to authorized users.

## Background

Variation in the host response to parasite infection depends on a variety of factors including age, gender, host genetics, pathogen strain, and environment. Age-associated increases in malaria severity are determined in part by the particularities of the age-specific immune milieu, making this an important and active area of research [1]. However, in addition to age-associated effects on infection, effects on the response to anti-parasite chemotherapy are not well understood, even though these effects may impede the global agenda for malaria elimination and eradication [2]. Therefore, a general lack of knowledge about age-associated differences in immune responses to *Plasmodium falciparum* infection and treatment constrains the development of protective anti-malarial vaccines and therapeutics for young children who, although initially at decreased risk for severe malaria during a primary infection, compared with adults, may be at increased risk for severe complications due to exposure history and/or immune dynamics [3–5].

In malaria-endemic regions, repeated exposure to parasites may generate adaptive immunity in some infant and young child populations as a mechanism for protection from severe disease, after the protection offered by maternal antibodies has waned [6–11]. However, age-associated changes in immune function may also contribute to improved immune responses in adults. Thus, recent studies have explored age-associated effects in order to understand the relative contribution of parasitological and host immunological effects on heterogeneity in the response to malaria infection.

Age-associated effects on the production of anti-*Plasmodium* antibodies against pre-erythrocytic and asexual blood stage antigens were recently reported by Ouédraogo et al. [12]. In addition, in children from Mozambique, significant associations were found between infant age and levels of IgG directed against merozoite-stage *Plasmodium* [13]. Furthermore, age-associated effects on B cell response magnitude [14] and post-treatment parasite clearance [15] have also been described. Whereas these studies focused on identifying age-associated differences in adaptive and antibody-related responses to parasite infection, this study focuses on age-associated differences in plasma cytokine and monocyte levels, since these may be critical for determining treatment efficacy in infant and young child populations.

Infants and young children face multiple barriers to overcoming malaria infection, including suboptimal innate immune responses to natural infection and poor anti-malarial treatment efficacy, which in some cases results in serious outcomes, such as severe malarial anaemia (SMA) or cerebral malaria (CM). Studies have shown that SMA and CM are driven by proinflammatory cytokine secretion and immunopathology, suggesting immunomodulation as a potential avenue for adjunctive therapy to prevent severe outcomes in infants [16–19]. Although SMA and CM have been a major focus of research in young children, the main interest of this study is to identify age-associated markers of treatment response in uncomplicated malaria (UM)—an area that is arguably less well studied and yet remains critical to understanding phenotypic variation in the majority of malaria-infected and treated young children. Therefore, in order to isolate age-associated effects on UM, and also to avoid exacerbation of disease among participants, individuals who showed evidence of severe anemia were excluded from the cohort.

In this study, young child and adult peripheral blood, collected during acute malaria infection and 4 weeks post-anti-malarial treatment, was examined to identify signatures of differential host responses to infection and treatment. Among the main findings, there were significantly higher plasma IL-10 and TNF levels, and nitric oxide metabolites, in young children compared with adults, regardless of treatment. IFN-*γ* and IL-12(p40) treatment responses also differed significantly based on age, in a gender-specific manner. In addition, several subjects (5 of 63) with apparent treatment failure, or reinfection. Thus, this work improves understanding of the age-associated response to malaria infection, implicating inflammatory differences in whole blood treatment responses on post-treatment infection resolution, and may contribute to the development of improved vaccines and therapies for paediatric populations.

## Methods

### Study population and sample collection

The area of this study, in Lilongwe, Malawi, is characterized primarily by unstable malaria transmission due to its relatively high elevation (1000–1100 m), and infections begin to peak during the rainy season. Subjects for this study were randomly selected from patients who tested positive for *Plasmodium falciparum* infection, February 1st, 2012 through May 22nd, 2012 at the Kamuzu Central Hospital (KCH) outpatient clinic in Lilongwe. A total of 34 infants and young children, henceforth “young children” (4–24 months) and 29 adults (19–70 years) were enrolled (Table 1). Informed written consent from adult participants and from parents of infant and young child participants was obtained during the first clinic visit. Enrollment in the study was voluntary and all infected patients received anti-malarial treatment independent of enrollment. The study was approved by the Institutional Review Board at UNC and the National Health Sciences Research Committee, under the oversight of the Ministry of Health, in Malawi. The institutional guidelines strictly adhere to the World Medical Association’s Declaration of Helsinki.

**Table 1:** Clinical characteristics of study participants

	Young children	Adults
Acute		
No. of participants	34	29
Age, years	0–2	19–70
Haemoglobin, g/dL blood	9.16 (2.06)	14.05 (2.53)
Parasite load, μL^−1^ blood (×10^4^)	7.08 (12.4)	1.10 (2.32)
Gametocytaemia, μL^−1^ blood		
Pfs16	36.97 (47.25)	44.19 (40.69)
Pfs25	2.74 (8.19)	0.517 (1.22)
Pfs230 (×10^5^)	2.24 (9.83)	15.6 (70.2)
% positive for anti-*Plasmodium* Ab	50.00	79.31
Post-treatment		
Haemoglobin, g/dL blood	10.76 (2.02)	13.03 (2.23)
Parasite load, μL^−1^ blood (×10^4^)	0.2589 (1.338)	0.0002 (0.0011)
% positive for anti-*Plasmodium* Ab	44.12	65.52

Where applicable, values are given as mean (1 sd)

Individuals who visited the hospital and whose clinical diagnosis was consistent with malaria were subsequently screened by a rapid diagnostic test (RDT, SD Bioline Malaria Ag P.f test, for qualitative detection of HRP-II antigen of *P. falciparum* in human whole blood, Cat # 05FK50) to determine malaria positivity, and then enrolled in the study (n = 63). Study participants were asked to donate a venous blood sample (young children: 3–5 mL; adults: 10 mL) at their first visit (V1; “acute pre-treatment”). Malaria infection was confirmed by microscopic examination of blood smears. Young children with severe malaria (haemoglobin < 8.0 g/dL and haematocrit < 18%) were excluded from the study to avoid the risk of exacerbating SMA. In addition, whole blood from participants was blotted and dried on Whatman 903™ protein saver cards (#10534612) for gametocytaemia analysis.

Infected participants were prescribed anti-malarial chemotherapy, which consisted of a first-line regimen of artemether-lumefantrine (AL), and were asked to return in 4–6 weeks for a second visit (V2; “post-treatment”) and blood sample collection. To ensure adherence to anti-malarial treatment and follow-up visits, patient tracing was employed. Patients who could not be adequately traced for follow up have no data for V2.

**Fig. 1:**
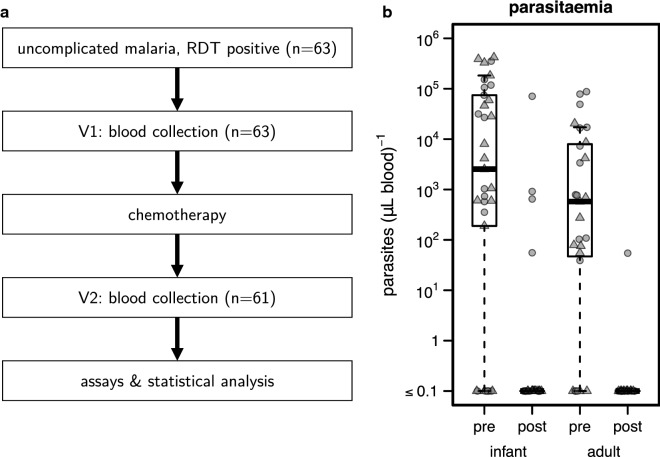
Young child age is associated with increases in blood-stage parasitaemia during acute infection, and incomplete parasite clearance post-treatment. **a** Study population and data collection. For each subject, the duration between V1 and V2 was 4 weeks. **b** Parasite load (parasites/μL in whole blood) was measured in infants and young children (“infant”) and adults by whole blood microscopy. Circular data points indicate female subjects, and triangles indicate male subjects

Subjects’ samples and clinical details were de-identified in Malawi. Age, gender, and parasitaemia of each patient were recorded with a corresponding unique patient ID code. Blood plasma was collected and stored at − 80 °C. Peripheral blood mononuclear cells (PBMCs) were isolated using Ficoll-Paque gradient separation and then frozen in 10% DMSO/90% fetal bovine serum (FBS) and stored in liquid nitrogen. De-identified samples, including blood plasma, PBMCs and dried blood spots, were shipped to the University of North Carolina at Chapel Hill for additional analysis. Details about the selection and phenotyping of study participants are summarized in (Fig. 1a).

### Parasite load

To determine the level of infection in all malaria-positive subjects, parasitaemia was quantified at the Kamuzu Central Hospital clinic in Malawi by light microscopy of thick blood smears at V1 and V2. All slides were read by two expert readers independently and mean values are used as phenotypes; in cases with data discordance, a third reader was assigned.

### Anti-malarial antibodies

Anti-malarial antibodies were assessed using a semi-quantitative human malaria antibody ELISA kit (IBL International Inc., Hamburg, Germany #RE58901), according to manufacturer’s protocol. From these results, the fraction of young child and adult participants who tested positive for malaria-specific antibodies (IgM or IgG) were calculated.

### Haemoglobin

Haemoglobin levels were measured in clinic, at V1 and V2, and are reported in g/dL.

### Nitric oxide

Plasma samples were deproteinated and NO metabolite levels (nitrite and nitrate) were quantified for V1 and V2 using the QuantiChrom™ nitric oxide assay kit (BioAssay Systems #D2NO-100). Quantification using OD was carried out according to the manufacturer’s protocol (PerkinElmer). Concentrations were based on absorbances normalized to the manufacturer’s standard and calculated via the Beer–Lambert law.

### Plasma cytokines

The following analytes were measured in the plasma, for V1 and V2, using the MILLIPLEX MAP Human Cytokine/Chemokine Magnetic Bead Panel/Immunology Multiplex Assay (EMD Millipore #HCYTOMAG-60 K): GM-CSF, IFN-*γ*, IL-10, IL-12(p40), IL-12(p70), sCD40L, IL-1*β*, IL-6, and TNF. Assays were performed according to the manufacturer protocols on a MagPix (Luminex) instrument at the UNC-Chapel Hill Center for AIDS Research (CFAR) HIV/STD Laboratory Core. Standard curves were fit and experimental concentrations determined from a 5-parameter weighted logistic model using the xPONENT^®^ software (v4.1.308.0).

### Monocyte and dendritic cell composition

Flow-cytometric analysis was performed to characterize myeloid dendritic cell (mDC) and monocyte (Mo) frequencies in PBMCs. All antibodies were purchased from BD Biosciences (San Jose, CA). Cells were stained according to BD protocols using the following mouse anti-human antibodies: CD3 (clone SP34-2), CD14 (clone M5E2), CD16 (clone 3G8), CD20 (clone 2H7), CD33 (clone P67.6), HLA-DR (clone G46.6), and CD11c (clone S-HCL-3). MDC frequencies were reported as percentage of mononuclear cells (MNC). Monocytes were further defined by gating as traditional monocytes (CD14^++^CD16^−^), inflammatory monocytes (CD14^++^CD16^+^) and patrolling monocytes (CD14^dim^ CD16^++^) (see Additional file 1: Figure S1). Samples were acquired on the LSR11 (BD; San Jose, CA) using FACS DIVA software and analysed with FlowJo (TreeStar, Inc., Ashland, OR).

### Statistical methods

Data was analysed in the statistical programming language R [20]. Responses were measured for each study participant, using peripheral blood samples collected at two time points: immediately after malaria diagnosis, at visit 1 (V1); and approximately 4 weeks after completing anti-malarial treatment, at visit 2 (V2). Some phenotypes were only measured at V1, and some were measured at both V1 and V2.

A zero-inflated Poisson (ZIP) regression model [21] (log link) was used to evaluate the effect of age and visit on microscopy-based parasite counts at V1 and V2. In brief, ZIP regression uses a two-component mixture model that simultaneously accounts for zero- and non-zero counts using a Poisson, as well as accounting for zero inflation using the binomial distribution (probit link), which is fit using maximum likelihood estimation via the R package pscl [22, 23].

To model effects of age on gametocytaemia, as measured from dried blood spots collected during V1 only, the exact Wilcoxon–Mann–Whitney two-sample rank-sum test was used via the R package coin [24], stratifying by gender. A two-sided *p*-value is reported.

To model effects of age and gender on anti-malarial antibody results (“negative”, “grey”, or “positive”) at V1 and V2, ordered logistic regression (a cumulative link model [25]), was used via the R package MASS [26].

For all additional blood analyte phenotypes that were measured at both V1 and V2, data was modelled using a rank-based nonparametric model that accommodates longitudinal data which is collected in a factorial design [27, 28]. The model is implemented in the R package nparLD [29]; ranks were contrasted between groups and used to calculate ANOVA-type statistics [30] according to the factors of interest, which were: age group (young child, adult), gender (male, female), visit (V1, V2), and their pairwise and three-way interactions. Among the study subjects, there were missing data points in at least one phenotype: for one individual on the first visit (V1), and for six individuals on the second visit (V2).

## Results

### Subjects

The study population was comprised of 63 enrolled subjects, including 34 young children < 2 years old (n_females_ = 16, n_males_ = 18), and 29 adults 18 years old (n_females_ = 16, n_males_ = 13). All enrolled subjects tested positive for malaria by RDT. Characteristics of the young child and adult participants are provided in Table 1 and Additional file 1: Table S1.

### Parasite load

To determine the effect of anti-malarial treatment on parasite burden in infected adults and young children, and to test for the effect of age and gender, parasite loads were quantified at V1 and V2 using microscopy of patients’ thick blood smears. During acute infection (V1), parasite loads were detected in 21 of the 27 adults measured (77.8%) and 25 of the 33 young children measured (75.8%). Among young children and adults with detectable parasite loads at V1, parasite counts were significantly higher on average (p < 10^−16^), by more than sixfold, in young children (9.35 × 10^4^ µL^−1^) compared with adults (1.40 × 10^4^ µL^−1^); in addition, a significant overall effect of age, and a significant age-by-gender interaction (both p < 2 × 10^−16^) were found. A significant overall zero-inflation intercept (*p* = 0.0225) was present, indicating the detection of excess zero (undetectable) counts in this data set, and these were unaffected by age or gender.

After anti-malarial treatment (V2), parasite counts decreased to undetectable in all but 5 female subjects who had residual detectable parasitaemia (1 adult, 4 young children). For 4 of these 5, parasite loads nevertheless decreased substantially from V1 to V2 (Fig. 1b).

### Haemoglobin

**Fig. 2:**
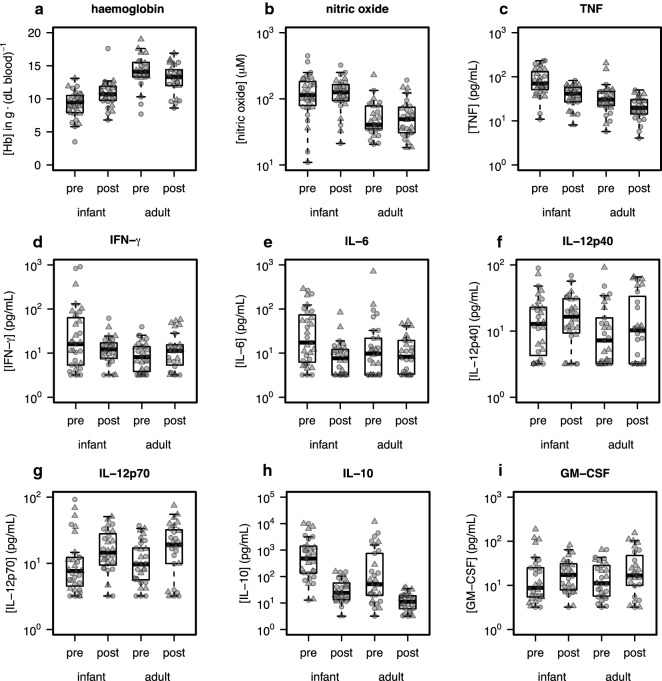
Blood markers in young children differ significantly from adults during acute infection, and respond differentially to antimalarial treatment. The concentrations of the following analytes were assayed, for adult and young child (“infant”) samples collected during acute infection and post-treatment (in pg/mL): **a** TNF, **b** IFN-*γ*, **c** IL-6, **d** IL-12(p40), **e** IL-12(p70), **f** IL-10, and **g** GM-CSF. Levels of **h** haemoglobin in whole blood (g/dL) and **i** nitric oxide metabolites (μM) in plasma were also analysed. Concentrations are presented for acute infection and post-treatment, and stratified by age group. Circles indicate females, and triangles indicate males

During their asexual stage, *Plasmodium* parasites digest haemoglobin (Hb) in erythrocytes as an energy source, resulting in the production of free heme. This free heme is toxic to the parasite, and is detoxified by parasite-mediated conversion to crystalline haematin (i.e. haemozoin) and is then stored in the parasite’s food vacuole. Effects of parasites on Hb levels in the blood may indicate differences in the physiology and composition of host erythrocytes. In order to determine the effect of gender, age, and anti-malarial treatment on Hb in study participants, Hb was measured at V1 and V2. A significant overall effect of age on Hb levels (higher in adults, *p *= 3.86 × 10^−15^), a significant main effect of gender (higher in females, *p* = 5.6 × 10^−3^), as well as a significant age:visit interaction effect (*p* = 3.14 × 10^−4^) were observed (Fig. 2a). Compared with the treatment response in adults, whose Hb levels were lower on V2 compared with V1, Hb levels in young children were higher on V2 compared with V1.

### Anti-malarial antibody response

During V1, half of all young children in the study (17 of 34 total; or 10 of 18 males and 7 of 16 females) had detectable anti-malarial antibodies, indicating prior exposure to malaria parasites or acquisition of maternal anti-malarial antibodies. This is in contrast with the 22 of 29 adults (75.9%; or 10 of 13 males and 12 of 16 females) who had detectable anti-malarial antibody at V1, suggesting increased parasite exposure or increased capacity for antibody production, resulting in increased antibody detectability, in adults compared with young children. Thus, a significant overall effect of age was observed (*p* = 0.0298), but no significant effects of gender or treatment. The detectability of anti-malarial antibodies was reduced to undetectable levels in five individuals between V1 and V2. Among these five individuals, two were adults (1 male, 1 female) and three were young children (2 males, 1 female). Only two subjects, both young children (1 male, 1 female), transitioned from no detectable anti-malarial antibody at V1 to detectable antibody at V2 (Additional file 1: Figure S4, Table S2).

### Nitric oxide

Nitric oxide (NO) is a molecular effector that is released by activated immune cells in their defense against parasite infection [31]. Increased plasma NO levels in adults and children have been associated with protection from malaria [32–34]. In order to determine whether age, gender, or treatment significantly affected NO levels in this population, NO metabolite levels in plasma were measured. Significant age-associated effects on NO metabolite levels were detected (*p* = 1.191 × 10^−10^). However, no significant overall effects of treatment on NO metabolite concentrations were detected. No significant gender-specific effect on NO metabolites was observed, although the variation in NO metabolites at both time points was substantially higher in young child females (sd_V1_ = 121.159, sd_V2_ = 82.213) than in young child males (sd_V1_ = 47.508, sd_V2_ = 49.970) (Fig. 2b).

### Plasma cytokines

To characterize the host immunological response to malaria infection and anti-malarial treatment, cytokine protein levels were measured using a MILLIPLEX panel of nine analytes [TNF, IFN-*γ*, IL-6, IL-12 (p40), IL-12 (p70), IL-10, GM-CSF, sCD40L, and IL-1*β*]. To model the data, a nonparametric, rank-based statistical framework developed for paired longitudinal measurements was used to ask if: (1) there are significant main effects of treatment (i.e., visit), gender, and/or age, and (2) if there are significant interaction effects (age:gender, age:visit, gender:visit, age:gender:visit) on plasma cytokine levels in the study population. The results are summarized below (Fig. 2c–i, Additional file 1: Table S3).

### Pro-inflammatory cytokines

A small, but highly significant, overall effect of visit on TNF levels was found (*p* = 1.282 × 10^−7^), where treatment (V2) was associated with reduced levels. A significant overall effect of age was observed (*p* = 1.200 × 10^−7^), where young children had higher overall levels compared with adults, and a marginally significant effect of gender (*p* = 4.569 × 10^−2^)—males had higher average levels of TNF in both age groups and time-points. A significant gender-specific effect on IFN-γ levels (*p* = 2.048 × 10^−2^), and an age:gender:visit interaction effect (*p* = 3.85 × 10^−3^) were found. IL-6 decreased significantly after treatment (*p* = 1.907 × 10^−2^). Even though no significant gender-based effects on IL-6 were detected, the discordant response observed between young child and adult samples in males contrasted with the similar response that was observed in both age groups in females. There was a significant overall treatment effect on IL-12(p70) levels (*p* = 3.483 × 10^−6^), where post-treatment levels were higher than during acute infection, and a nearly significant gender effect (*p* = 1.291 × 10^−2^) where males had slightly higher values at both time points and in both age groups. There was no overall effect of age on IL-12(p40) levels, however, in males, there appeared to be a treatment effect in adults only, with higher IL-12(p40) levels after treatment, and in females, there appeared to be a treatment effect in young children only, with higher IL-12(p40) levels after treatment. This manifested as a marginal age:gender:treat effect (*p* = 3.475 × 10^−2^).

Observed levels of IL-1*β* were often below detectable limit, and the levels of sCD40L were often above the detectable range, making their quantification highly uncertain, and leading to the exclusion of those cytokine measurements from the analysis.

### Anti-inflammatory cytokine and growth factor

A significant effect of visit (treatment) on plasma levels of IL-10 was observed (*p* = 2.566 × 10^−15^), where post-treatment levels were substantially lower than during acute infection. A significant effect of age on IL-10 was found, where young children had significantly higher levels than adults at both time points (*p* = 3.305 × 10^−7^). A small but significant effect of treatment on levels of GM-CSF in the plasma was found (*p* = 1.151 × 10^−3^), where post-treatment individuals had slightly elevated GM-CSF, regardless of age group. Males trended toward higher mean values across the two-time points and ages.

### Treatment failure or reinfection

Five individuals remained parasitaemic even after treatment, indicating treatment failure, problems with adherence or dosing, and/or reinfection by V2 (Additional file 1: Figure S5). Among the five, parasite levels were reduced by only 5% in a single female infant, and by > 97% in the remaining 4 individuals. All five individuals had lower plasma IL-10 and TNF on V2 compared with V1, similar to the general effect across all study participants. However, notable among most of these subjects is the substantial decrease in IL-6 to very low levels on V2.

### Plasma cytokine ratios

**Fig. 3:**
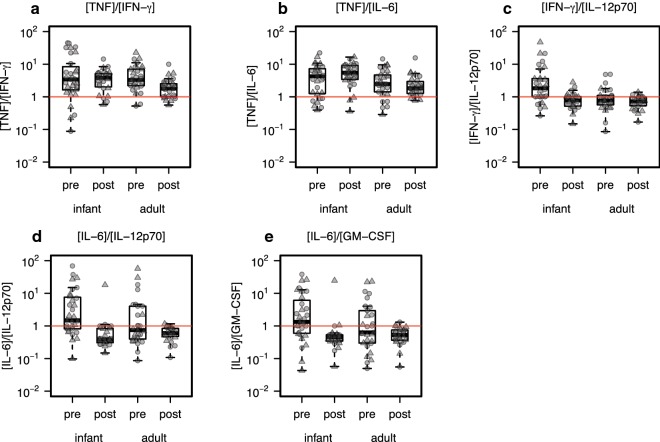
Treatment response of blood analyte ratios is modified or reversed in young children compared with adults. Analyte ratios for which a significant treatment response interaction with age was discovered (5 of 15 tested) are presented as proportions, for: **a** TNF/IFN-*γ*, **b** TNF/IL-6, **c** IFN-*γ*/IL-12(p70), **d** IL-6/IL-12(p70), and (E) IL-6/GM-CSF. The horizontal line demarks a ratio of 1:1; “infant” = infants and young children

The ratios of distinct plasma analytes, many of which simultaneously compete to modify the plasma immune milieu, may more precisely characterize the immune landscape at different levels of treatment, age, or gender. The plasma cytokines, TNF, IFN-*γ*, IL-6, IL-12(p70), IL-10, and GM-CSF, were examined, consisting of 15 pairwise analyte combinations, and effects of age, gender, and visit on their proportions were analyzed. Significant overall effects of age were found in 10 of 15 of the proportions examined. In contrast to effects on individual analyte levels, no overall gender-specific effects on analyte ratios were found. Significant overall effects of treatment (visit) on 13 of 15 analyte proportions were observed, and significant age effects on treatment response for five of 15 proportions, with the most significant effects observed on IL-6/IL-12(p70) treatment response (*p* = 1.385 × 10^−4^) and IL-6/GM-CSF treatment response (*p* = 8.994 × 10^−4^), where age reversed the direction of the treatment response in both cases (Fig. 3, Additional file 1: Table S4). The most significant gender-dependent age effects on treatment response were observed for IFN-*γ*/IL-12(p70) (*p* = 8.849 × 10^−4^) and IFN-*γ*/GM-CSF (*p* = 9.116 × 10^−4^).

### Monocyte and dendritic cell composition

**Fig. 4:**
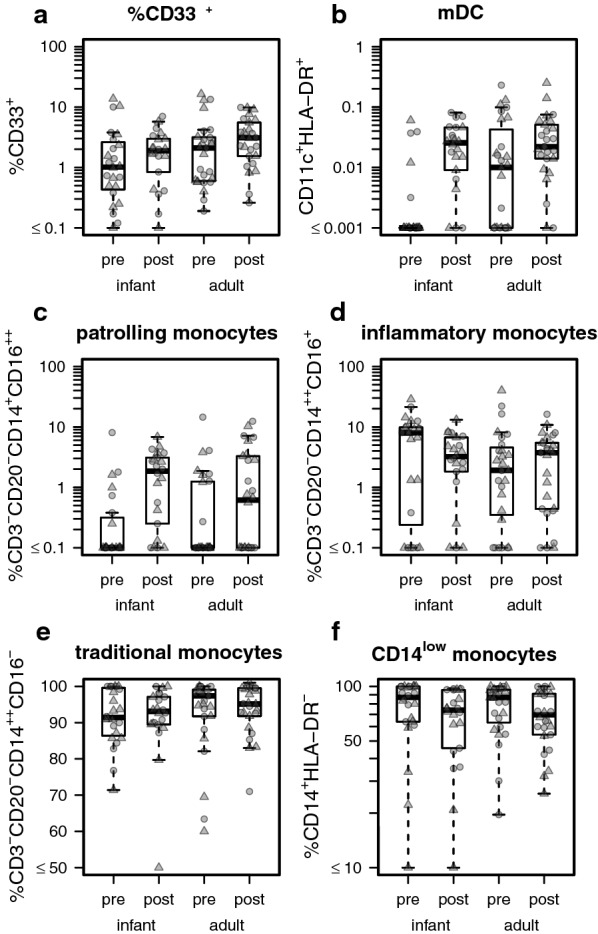
The composition of myeloid DCs, patrolling monocytes, and CD14^low^ monocytes differs based on age and/or visit. The percent composition of **a** CD33^+^ cells, **b** mDCs among all viable PBMCs is shown. The percent composition of **c** patrolling, **d** inflammatory, and **e** traditional monocyte subsets, as a fraction of all monocytes, as well as **f** the percent of CD14^low^ monocytes, as a percentage of all CD16^−^ monocytes, are shown. Percentages are stratified by age group (“infant” = infants and young children) and visit

Functional differences in immune responses and inflammatory signaling between individuals may be mediated by differences in the overall composition of monocytes and monocyte-derived cellular populations circulating in the blood. No significant difference in percentages of CD33^+^ cells based on age, gender, or visit/treatment were observed, however there was an overall trend for higher percentages observed in the second visit compared with the first, and for higher levels in adults compared with young children (Fig. 4a). The proportion of myeloid dendritic cells (mDCs) among all PMBCs, while very small (often < 0.1%), was found to be significantly higher post-treatment than during acute infection in all groups (*p* = 6.032 × 10^−8^). In addition, nearly significant effects were observed for age (*p* = 4.665 × 10^−2^) and an age:visit interactions (*p* = 4.282 × 10^−2^), mostly due to lower mDC levels in young children vs. adults during the acute visit (similar levels of post-treatment mDCs) (Fig. 4b).

Traditional, classical and patrolling monocytes serve different roles in pathogen surveillance, effector functions, and disease pathogenesis [35]. A significant treatment effect on patrolling monocytes was observed (*p* = 1.168 × 10^−5^), where levels increased significantly post-treatment in both young child and adult populations (Fig. 4c). Although not significant, it appeared that age changed the direction of the treatment response for both inflammatory (Fig. 4d) and traditional monocytes (Fig. 4e). A significant treatment effect was found on the frequency of CD14^low^ monocytes (*p* = 1.648 × 10^−2^) as a percentage of the total CD16^−^ monocytes (Fig. 4f).

**Fig. 5:**
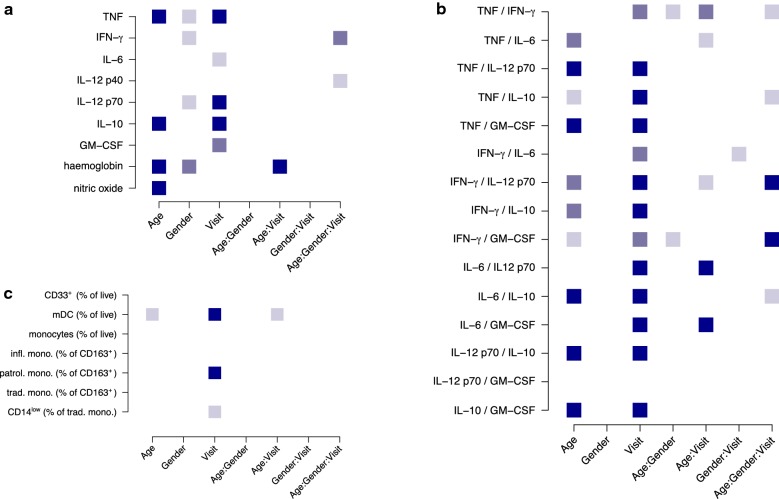
Significant factors and interactions on blood analytes, analyte ratios, and cellular phenotypes identified in this study. Nominal *p*-values for factors identified by nonparametric analysis of blood analytes (**a**), analyte proportions (**b**), and cellular data (**c**) are indicated by colour (light blue: *p* < 0:05; medium blue: *p* < 0:01; dark blue: *p* < 0:001)

A summary of *p*-values for age, gender, visit, and interaction effects for all analyte, analyte ratio, and cellular *p*-values is included in Fig. 5.

### Within-group age-associated effects on analyte levels

Additional blood analyte heterogeneity within-group, adult or young child, may be caused by age-associated effects that are not captured by the binary coding of age used in the main analysis. To identify continuous rather than categorical age effects, a linear model was used, fitting age (in years for adults, or fraction of years for young children) and age-by-gender effects for adults and young children separately, at each treatment timepoint, and fitting the same effects for the log_2_-fold change between acute and post-treatment visits. Although no significant effects on the treatment response (log_2_-fold change) were found, significant within-group age effects were identified at both visit 1 and visit 2.

**Fig. 6:**
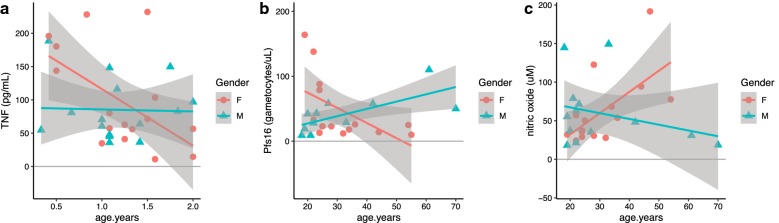
Continuous age is associated with differences in host and parasite factors in both young child and adult populations. Continuous age effects and age-by-gender interaction effects are shown for **a** TNF at V1 for young children, **b** *Pfs16* levels at V1 for adults, and **c** NO metabolite levels at V2 for adults. Age is presented in years

At visit 1, significant within-group age effects on young child TNF were observed (ANOVA-like *p* = 0.008, decrease with age, appears to be driven by females) (Fig. 6a), and on adult GM-CSF (*p* = 0.032, increase with age), and adult *Pfs16* (*p* = 0.00976, decrease with age), including substantial effects of age (*p* = 0.0032) and age-by-gender interaction (*p* = 0.0027) (Fig. 6b).

At visit 2, significant within-group age effects on adult nitric oxide metabolites (*p* = 0.014, increase with age), including substantial effects of age (*p* = 0.017) and age-by-gender interaction (*p* = 0.0093) were observed (Fig. 6c).

## Discussion

In this study, age-associated differences in the anti-malarial treatment response were evaluated in adults and young children acutely infected with *P. falciparum*, the predominant malaria parasite in southern Africa. Evidence was provided for substantial widespread differences in immune regulatory factors and cellular effectors between adults and young children who are infected with *P. falciparum* and subsequently treated, suggesting that age-associated factors may interfere with host-intrinsic anti-parasite immunity, with repercussions for anti-parasite chemotherapy.

Substantial effects of young age on blood-stage parasitaemia and gametocytaemia were observed, as well as a greater risk of recrudescence or reinfection. Young children exhibited significantly higher parasite loads during the first clinical visit, and higher levels of (*Pfs25*-expressing) mature gametocytes, reflecting potential differences in biology, disease presentation and/or healthcare seeking. In addition, blood marker levels were significantly different in young children compared with adults during acute infection, and changes in these levels in response to treatment also differed. When considering co-variation of blood analytes, in the form of cytokine ratios, young child age was found to modify or reverse the effects of treatment for some cytokine pairs. Age-associated differences in the treatment response of myeloid DCs were also observed. Finally, within each age group, continuous age effects, and age-by-gender effects, contributed to phenotypic differences observed at V1 and V2, sometimes transgressing the observed group-wise age effects, shedding light on the complexity of immune development at long-term and short-term time scales.

It is clear that age plays a role in the eventual outcomes of complicated and uncomplicated malaria treatment. Prior studies have identified age-associated treatment effects on infection recurrence [36] and treatment failure for a number of anti-malarial drugs [37–42]. Even so, the relationship between age and malaria infection severity is complex. Although it has been shown that, among naive individuals, adults are more susceptible to severe malaria than infants and young children [43], and that increasing age within adults leads to increasing susceptibility to severe malaria [44], over time, older children, likely due to exposure, exhibit differentiated tolerance to malaria infection [45]. In addition, infected infants and young children are more likely to have severe anemia, metabolic acidosis, cerebral malaria, and convulsions [46]. Variation in the host immunological response to infection and treatment may underlie variability in clinical outcomes, and infants and young children, likely due in large part to parasite exposure history, are especially at risk for adverse outcomes even when adequate anti-malarials are available.

A number of factors are important for anti-malarial therapeutic response, and may play a role in age-associated differences in outcomes, including: drug resistance, drug dosing, and drug absorption, as well as effects of immunity of the human host [42]. Recent analyses have indicated that current recommended doses in young children may be too low [47]; these, and other age-related effects of immune exposure and immune development may contribute to treatment response differences between young children and adults.

Transplacentally-transferred antibody levels decrease over time after birth [48]. Total anti-*Plasmodium* IgG and IgM levels were detectable at a lower frequency in young children compared with adults, potentially conferring differential protection from pathology in infected individuals. Even so, a substantial proportion of young children (50% at V1 and 44% at V2) tested positive, suggesting high rates of prior exposure in young children and/or retention of substantial detectable maternal antibody. In addition, the presence of samples that converted to seronegative after treatment suggests that the antibodies chosen for detection are for some reason not durable/long-lasting. Even so, the assay that was used is unable to distinguish acute from prior exposure. In Ghanaian and Gambian children, antibody duration differences were previously associated with differences in short- and long-lived antibody secreting cells (ASCs) [49]. Antibodies targeting alternative parasite antigens may have different temporal kinetics, and may be useful for estimation of prior exposure in children [50]. As such, the malaria Ab seropositivity reported here provides a minimum estimate of the proportion of individuals that have had prior exposure.

Studies over the last several decades have shown that nitric oxide (NO) levels are associated with protection from malarial disease [51–55], and prior work has reported age-associated effects on NO metabolites during malaria infection [56]. The results presented here suggest that NO metabolite levels are upregulated in young children compared with adults, however, it is important to note that these measures did not change between V1 and V2, and they did not correlate with parasitaemia as other studies have found [57].

In a study of age effects on T cell cytokine production, IFN-*γ*-producing CD4+ and CD8+ T cells were shown to be higher in adults compared with infants [58]. In a longitudinal study of infected children in Gabon, TNF and IFN-*γ* were found to be positively correlated with parasitaemia, and there was an associated decline in the levels of these cytokines across groups of increasing age [59]. In a study of acute-phase malaria in young children in Gabon, patients with severe malaria had significantly lower IL-12(p40/p70) and IFN-γ compared to matched controls with mild malaria; in addition, TNF and IL-10 were significantly higher in severe malarial cases [60]. In contrast, in a prospective study, increased IFN-*γ* and TNF production, in parasite-induced whole blood cultures, was found to be associated with reduced risk of fever and/or clinical disease [61].

In this study, young children had overall significantly higher TNF and IL-10 compared with adults, and no overall effect of age on IFN-*γ*, IL-12(p70) or IL-12(p40), suggesting that infant profiles in this cohort may more closely represent an inflammatory environment signifying severe disease risk, although the source and context of cytokine secretion may determine the relevance to clinical outcomes. Notably, a weak signal of a gender-specific age-associated treatment effect reversal was observed, with a trend towards IFN-*γ* levels decreasing after treatment in young children, and increasing after treatment in adults (Fig. 5).

In a study of malaria infection of children in Mali, severe malaria cases, in comparison with matched healthy controls, showed higher levels of IL-6, IL-10, TNF, IL-12(p70), and IL-6 and IL-10 were higher in severe cases in comparison with matched uncomplicated malaria controls [62]. In this study, no age-associated effects on IL-6 or IL-12(p70) were found, but there was an overall age effect on IL-10 (higher in young children vs. adults, regardless of visit timepoint), which is also consistent with a more severe disease risk in the young child age group compared with adults. Furthermore, in this study, an apparent inverse regulatory relationship of IL-10 and IL-12(p70)/IL-12(p40) was observed, as previously reported [63], although overall age-associated effects were more apparent in IL-10 levels compared with IL-12, for which they were not detected.

The peripheral blood monocyte response to malaria infection may affect the course of the host response through antibody-dependent cellular inhibition (ADCI) and opsonic phagocytosis of parasites and merozoites/infected RBCs [64–66]. In addition, monocyte-derived cytokines/chemokines may contribute to malaria severity [67]. Experimental infection studies have shown that overall monocyte levels increase during blood-stage infection, and DC levels are mostly stable [68]. In this study, in contrast, regardless of age group, treatment resulted in no significant increase in overall monocytes, although significant increases in patrolling monocytes were observed; and myeloid dendritic cells also increased after treatment, regardless of age (Fig. 4). Low monocyte counts in malaria-infected children have been associated with increased risk of convulsions and increased mortality [69]. In a study of infants and adults in Thailand, low peripheral blood monocyte counts were significantly associated with high parasite loads [70]. Despite these protective monocyte associations, in a study of severe and uncomplicated malaria in children under 5 in Uganda, increased pigmented monocyte counts, assessed by blood smears, have been associated with increased infection severity [71]. In a multi-year cross-sectional study of children in the Kilifi District in Kenya, malaria episode risk increased with higher monocyte counts [72]. In addition, although not explored here, differences in monocyte function, including inflammatory/regulatory cytokine production and cell surface marker expression, are also important correlates of disease severity in children [73]. In this study, no strong age-specific associations with overall counts or subsets of monocytes were found, but trends were observed toward age-associated reversals of the treatment response on inflammatory and traditional monocyte composition (Fig. 4). Resolution of acute infection was found to correspond with an overall decrease in inflammatory monocytes, in agreement with a recent study of children 1–10 years old in Kenya [74].

There are a number of limitations that are present in this study which should be addressed in future work. Regarding drug treatment, with the data that was collected, true treatment failures (recrudescence) cannot be distinguished from parasite reinfection. Differences in drug dosing between infants and adults may account for some age-associated effects: young children may have altered pharmacokinetics, tend to vomit doses of medicine, and/or have differential adherence to treatment compared with adults. Although levels of compliance can be improved by various adjustments to study protocols [75], direct supervision is the only way to ensure treatments are faithfully administered. It is not known whether the parasite loads observed on V2 were due to new infections. To distinguish recrudescence from re-infection, genotyping of allelic variants of genes encoding *Plasmodium* merozoite surface proteins, *msp1* and *msp2*, or multi-SNP “barcodes”, is recommended [76]. In addition, to better understand the dynamics and efficacy of treatment, it would be informative to evaluate early parasite clearance data, i.e. measurements taken within the first few hours of chemotherapy. This study was also limited in its ability to assess parasitaemia at low levels, which would help reconcile any discordance between RDT- and microscopy-based diagnostics. This can be addressed, for instance, by using qPCR-based molecular quantification of asexual parasitaemia [77, 78]. There was a lack of power to detect many effects, due to the small sample size in both the adult and infant groups. Finally, this study is also limited in its ability to uncover covariates that may mediate the observed age-associated differences. To address this, future studies should aim to collect additional clinical data, such as: respiratory rates, co-morbidities, hypoglycaemia, acidosis, dietary nitrate ingestion, history of previous exposure to malaria, and the duration of fever/illness.

## Conclusion

In summary, this work shows that there are signatures from peripheral blood biomarkers that may indicate or contribute to immune response differences young children and adults in a region of seasonal malaria. These differences in inflammatory cytokines and peripheral blood cell populations may drive the clinical differences observed in disease risk between young children and adults, and furthermore gender effects may play a modifying role. Finally, the lack of efficacy of anti-malarial therapy in some individuals, caused by incomplete clearance or repeat infection, may be a function of cytokine dysregulation in the host response, and identification of the regulatory pathways that are altered will be critical to improving chemotherapy outcomes in young children.

## Additional file

**Additional file 1.** Additional methods. **Figure S1.** Manual gating strategy for quantifying monocyte subsets. **Figure S2.** Young age is associated with increases in blood-stage mature gametocytaemia during acute infection. **Figure S3.** Levels of gametocytaemia are similar in subjects with and without microscopically-detected parasitaemia. **Figure S4.** *Plasmodium*-specific antibody levels are higher in adults compared with young children regardless of visit. **Figure S5.** Correspondence between acute and post-treatment phenotypes. **Figure S6.** Q–Q plots of analyte phenotypes before and after Box-Cox-assisted power transformation. **Table S1.** Clinical characteristics of study participants (mean, interquartile range). **Table S2.** Results from anti-malarial antibody detection. **Table S3.** Table of *p*-values for main effects and interaction effects on blood analytes and cellular phenotypes, analysed using a nonparametric longitudinal model. **Table S4.** Table of *p*-values for main effects and interaction effects on blood analyte ratios. **Table S5.** Table of *p*-values for main effects and interaction effects on blood analytes, analysed using a linear mixed model.

## Data Availability

The data file and analysis scripts are available in the malariaInfantStudy repository on GitHub at https://github.com/mauriziopaul/malariaInfantStudy, with a static version at 10.5281/zenodo.2583961.
