# Supplementary material for: Signatures of divergent anti-malarial treatment responses in peripheral blood from adults and young children in Malawi
Source: Malar J. 2019 Jun 24;18:205. doi: 10.1186/s12936-019-2842-7 (PMC6591936; doi:10.1186/s12936-019-2842-7)
Supplement: Supplementary file 1 — Additional file 1. Additional methods. Figure S1. Manual gating strategy for quantifying monocyte subsets. Figure S2. Young age is associated with increases in blood-stage mature gametocytaemia during acute infection. Figure S3. Levels of gametocytaemia are similar in subjects with and without microscopically-detected parasitaemia. Figure S4. Plasmodium-specific antibody levels are higher in adults compared with young children regardless of visit. Figure S5. Correspondence between acute and post-treatment phenotypes. Figure S6. Q–Q plots of analyte phenotypes before and after Box-Cox-assisted power transformation. Table S1. Clinical characteristics of study participants (mean, interquartile range). Table S2. Results from anti-malarial antibody detection. Table S3. Table of p-values for main effects and interaction effects on blood analytes and cellular phenotypes, analysed using a nonparametric longitudinal model. Table S4. Table of p-values for main effects and interaction effects on blood analyte ratios. Table S5. Table of p-values for main effects and interaction effects on blood analytes, analysed using a linear mixed model. [file 12936_2019_2842_MOESM1_ESM.docx]

**Additional Materials for Malaria Infant Study**

**Additional Methods**

*Additional Experimental Methods*

*Inclusion and exclusion criteria*

The inclusion criteria required: age within 2-24 months for young children and 18 years or older for adults; individuals seen at the Kamuzu Central Hospital (KCH) outpatient clinic in Lilongwe, Malawi. The exclusion criteria included: any patient who has received a malaria vaccination in the past; any patient who exhibits signs of severe complications of malaria, including signs of seizure, altered mental status, coma, sepsis or systemic inflammatory response syndrome. Furthermore, among the paediatric patients, infants and young children with severe anaemia (haemoglobin <8.0 g/dL and haematocrit < 18%) were excluded at the discretion of the attending physician/nurse if a blood sample of 3 mL would be deemed unacceptable because it could present a potential associated health risk for the infant.

*Anti-malarial chemotherapy*

Patients were given a 3-day course of artemether–lumefantrine combination tablets (20 mg artemether plus 120 mg lumefantrine per tablet), for 6 doses in total taken at 0, 8, 24, 36, 48 and 60 hours. The doses were determined by body weight as follows: 5-14 kg (1 tablet/dose); 15–24 kg (2 tablets/dose); 25–34 kg (3 tablets/dose); > 35 kg (4 tablets/dose).

*Stage-specific gametocytaemia*

To detect mature parasite infection based on estimated gametocyte load, quantitative real-time PCR (qRT-PCR) was used. Total DNA and total RNA were isolated from subjects’ dried blood spots at V1 using nucleic acid purification kits (Norgen Biotek #35300, #36000), and cDNAs were used in qRT-PCR assays [[79](#page17), [80](#page17)]. These cDNAs were used to measure RNA expression levels of *P. falciparum* gametocyte-specific genes, including *Pfs25* (mature gametocyte), *Pfs16* (early gametocyte) [[81](#page17)], and *Pfs230*, which encodes a gametocyte antigen important for eliciting host antibodies that inhibit parasite transmission to a mosquito host [[82](#page17), [83](#page17)].

**Additional statistical methods**

*Parametric analysis of analytes*

^^In order to properly transform the data prior to using a linear mixed model, heteroskedasticity of the residuals was resolved using a power transform based on a Box-Cox analysis. First, the data were fit using a linear model:

(1)

where the values for *ε* are i.i.d. normal. This fit was then used to determine the value of with the maximum log likelihood, where represents the exponent and divisor in the following data transform:

(2)

where log is the natural log, according to [[84](#page17)], implemented in the R package MASS [[26](#page14)]. After finding approximate optimal values of lambda, the transforms used for each phenotypes are as follows, where fractions represent the values of lambda used, “none” represents no transformation, and “log” is the natural log transformation:

The quantile-quantile (Q-Q) plots are shown before and after the power transformations are applied in Figure [S6](#page23). A linear mixed model was then applied to the transformed data, according to the same model in equation (1), but adding individual-level random slopes (for visit).

**Additional Results**

*Stage-specific gametocytaemia*

In the human host, *Plasmodium falciparum* traverses through several life stages. During bloodfeeding at the dermis of a naive host, an infected female *Anopheles* mosquito transmits parasites to the host bloodstream as sporozoites. They mature into schizonts in the liver, rupture from liver cells as merozoites, and grow as ring-stage trophozoites which then enter a schizont-merozoite-trophozoite cycle [[85](#page17), [86](#page17)]. A proportion of blood-stage parasites develop into gametocytes, which are the haploid, sexual stage parasites that can subsequently be transmitted to a new female mosquito during bloodfeeding. Whereas it is blood stage parasites that primarily drive clinical disease, gametocytes are important for human-to-mosquito transmission.

In order to determine the effect of age on stage-specific gametocyte levels in patients’ blood at V1, *P. falciparum* gametocyte levels in subjects’ blood were estimated using qRT-PCR. Gametocyte stage-specific primers were used to quantify gene expression of *Pfs16*, *Pfs25* and *Pfs230* from cDNAs prepared from dried blood spots taken during V1. These quantities are not significantly correlated (*p* > 0.08), indicating that they are likely marking different gametocyte populations in this data set. Whereas, using the *Pfs16* (Figure S2A) and *Pfs230* (Figure S2C) primers, the estimated number of gametocytes did not differ significantly between age groups, young children had significantly higher levels of *Pfs25*-expressing gametocytes (median 0.465 gametocytes/µL) compared with adults (0.255/µL) (*p* = 0.00685) (Figure S2B). No gender-based differences in gene expression for *Pfs16*, *Pfs25* or *Pfs230* were observed.

*Gametocytaemia detected in individuals with no microscopically-detected parasitaemia at V1*

A number of individuals in the study tested positive by RDT and exhibited clinical symptoms of malaria but nevertheless had no microscopically detectable asexual parasitaemia in their blood. However, levels of gametocytaemia at V1 did not differ between individuals who had parasite counts = 0 and those who had parasite counts > 0 (Figure [S3](#page20)), controlling for age and gender, suggesting that active malaria infection was ongoing in these individuals despite the lack of detection of asexual parasites.

*Results for sCD40L and IL-1*

Soluble CD40 ligand (sCD40L, also known as sCD154) has been shown to induce DC maturation following *Plasmodium* infection in vitro, which can result in release of TNF and IL-12(p70) [[87](#page17)]. There was a marginal visit effect (*p* = 8.783×10^-3^), with higher levels post-treatment, as well as a marginal gender-specific effect on sCD40L (*p* = 6.734×10^-3^), with higher overall levels in males. However, the distribution of phenotypes was near the upper detection limit for this cytokine, which may have reduced the power of the assay to detect differences based on treatment, age, and gender. IL-1*β* levels in plasma increased significantly after treatment (*p* = 9.162×10^-4^), and there were no other significant effects. This cytokine was at the lower limit of detection, which may have also reduced power to detect significant effects.

*Linear mixed model analysis of analytes*

Subtle differences in the main results are expected based on the model selected. To determine whether the findings are sensitive to model type and specification, a linear mixed model was applied in addition to the nonparametric model reported in the main results. The majority (9 of 16) of the significant effects identified by the nonparametric model, presented in Figure 6, were also found using a LMM, as described above (Supplemental Table [S5](#page38)). Using ANOVA on the LMM (package: lmerTest), three additional significant effects were found for IL-6 (gender, *p* = 0.041) and IL-10 (age, *p* = 0.0096; age-by-gender, *p* = 0.020) [[88](#page17)].

**List of Additional Figures**

S1 Manual gating strategy for quantifying monocyte subsets

S2 Young age is associated with increases in blood-stage mature gametocytaemia during acute infection

S3 Levels of gametocytaemia are similar in subjects with and without microscopically-detected parasitaemia

S4 *Plasmodium*-specific antibody levels are higher in adults compared with young children regardless of visit

S5 Correspondence between acute and post-treatment phenotypes

S6 Q-Q plots of analyte phenotypes before and after Box-Cox-assisted power transformation

**List of Additional Tables**

S1 Clinical characteristics of study participants (mean, interquartile range)

S2 Results from anti-malarial antibody detection

S3 Table of *p*-values for main effects and interaction effects on blood analytes and cellular phenotypes, analysed using a nonparametric longitudinal model

S4 Table of *p*-values for main effects and interaction effects on blood analyte ratios

S5 Table of *p*-values for main effects and interaction effects on blood analytes, analysed using a linear mixed model

**Figure S1**. Manual gating strategy for quantifying monocyte subsets. (A) Monocyte cell counts and percentages are derived for CD14^++^CD16^—^ traditional monocytes, as designated by quadrant (a), CD14^++^CD16^+^ inflammatory monocytes, as designated in quadrant (b), and CD14^+^, CD16^++^ patrolling monocytes, as designated in quadrant (c), shown here for an example healthy, uninfected adult. A population of monocytes with intermediate CD14 brightness is designated in quadrant (d). (B) Example of flow cytometric phenotypic characterization of monocyte subsets in a *Plasmodium*-infected young children (“infant”) and adults is shown before and after treatment.

**Figure S2**: Young age is associated with increases in blood-stage mature gametocytaemia during acute infection. Gametocytaemia was measured in young children (“infant”) and adults using quantitative real-time PCR on cDNAs prepared from dried blood spot RNA, and gametocyte quantification was based on stage-specific expression of the following *Plasmodium falciparum* genes during acute infection: *Pfs16* (early gametocyte) (B); *Pfs25* (mature gametocyte) (C); and *Pfs230* (candidate gametocyte gene for transmission-blocking vaccine) (D), presented as gametocytes/*μ*L blood. Circular data points indicate female subjects, and triangles indicate male subjects.

**Figure S3**. Levels of gametocytaemia are similar in subjects with and without detected parasitaemia. The relationship of detectable parasitaemia, by microscopy, and gametocytaemia (gametocytes/*μ*L blood) in study subjects, as measured by *Pfs16* (A); *Pfs25* (mature gametocyte) (B); and *Pfs230* (C). No significant differences were detected, using the Wilcoxon-Mann-Whitney two-sample rank-sum test, controlling for age and gender.

**Figure S4**. *Plasmodium*-specific antibody levels higher in adults compared with young children regardless of visit. (A) The test result proportions for overall circulating anti-malarial IgG and IgM, measured by ELISA test of blood samples, are shown. The change in test result status between visit 1 and 2 is shown for (B) young children (“infant”) and (C) adults. Colours indicate positive (red), uncertain (grey), and negative (yellow) test results; subjects for which tests were not completed (ND) are shown in white.

**Figure S5**. Correspondence between acute and post-treatment phenotypes. The log(base=10) values of phenotypes from Figure 1B and Figure 2 are presented, with values at Visit 1 (acute) provided along the *x*-axis, and values for Visit 2 (post-treatment) along the *y*-axis. The black line in each figure indicates *y* = *x*. Circles indicate females and triangles indicate males. Red indicates young children and black indicates adults. The five darker coloured dots in each plot indicate phenotypes for individuals with apparent treatment failure.

**Figure S6.** Q-Q plots of analyte phenotypes (A) before and (B) after Box-Cox-assisted power transformation.

**Table S1**: Clinical characteristics of study participants (median, interquartile range).

**Table S2**: Results from anti-malarial antibody detection. Values indicate the proportion of samples within each age-visit group that received a given result for total Ig (IgG and IgM), where: “grey” indicates that the sample result was indeterminate and “N/A” indicates that the sample or result is missing.

**Table S3**: Table of *p*-values for main effects and interaction effects on blood analytes and cellular phenotypes, analysed using a nonparametric longitudinal model.

**Table S4**: Table of *p*-values for main effects and interaction effects on blood analyte ratios.

**Table S5**: Table of *p*-values for main effects and interaction effects on blood analytes, analysed using a linear mixed model. *P*-values for model intercepts were all highly significant (ranging in scale from 10^-17^ to 10^-31^) and were omitted from this table.

References

79. Kim HS, Smithies O. Recombinant fragment assay for gene targetting based on the polymerase chain reaction. Nucleic Acids Res. 1988;16:8887–903.

80. Wampler R, Timinao L, Beck HP, Soulama I, Tiono AB, Siba P, et al. Novel genotyping tools for investigating transmission dynamics of *Plasmodium falciparum*. J Infect Dis. 2014;210:1188–97.

81. Schneider P, Schoone G, Schallig H, Verhage D, Telgt D, Eling W, et al. Quantification of *Plasmodium falciparum* gametocytes in differential stages of development by quantitative nucleic acid sequence-based amplification. Mol Biochem Parasitol. 2004;137:35–41.

82. Williamson KC, Keister DB, Muratova O, Kaslow DC. Recombinant Pfs230, a *Plasmodium falciparum* gametocyte protein, induces antisera that reduce the infectivity of *Plasmodium falciparum* to mosquitoes. Mol Biochem Parasitol. 1995;75:33–42.

83. Stone WJ, Campo JJ, Ouédraogo AL, Meerstein-Kessel L, Morlais I, Da D, et al. Unravelling the immune signature of *Plasmodium falciparum* transmission-reducing immunity. Nat Commun. 2018;9:558.

84. Box GEP, Cox DR. An analysis of transformations. J R Stat Soc Series B Stat Methodol. 1964;26:211–52.

85. Ménard R, Tavares J, Cockburn I, Markus M, Zavala F, Amino R. Looking under the skin: the first steps in malarial infection and immunity. Nat Rev Microbiol. 2013;11:701–12.

86. Crompton PD, Moebius J, Portugal S, Waisberg M, Hart G, Garver LS, et al. Malaria immunity in man and mosquito: insights into unsolved mysteries of a deadly infectious disease. Annu Rev Immunol. 2014;32:157–87.

87. Mukherjee P, Chauhan VS. *Plasmodium falciparum*-free merozoites and infected RBCs distinctly affect soluble CD40 ligand-mediated maturation of immature monocyte-derived dendritic cells. J Leukoc Biol. 2008;84:244–54.

88. Kuznetsova A, Brockhof PB, Christensen RHB. lmerTest Package: tests in linear mixed effects models. J Stat Softw. 2017;82:1–26.
